# Changes in hemodialysis catheter management after introduction of the end-stage renal disease prospective payment system

**DOI:** 10.1186/s12882-020-02222-9

**Published:** 2021-01-06

**Authors:** Nicholas S. Roetker, Haifeng Guo, Marquita R. Decker-Palmer, Yi Peng, James B. Wetmore

**Affiliations:** 1 Chronic Disease Research Group, Hennepin Healthcare Research Institute, 701 Park Ave., Suite S2.100, Minneapolis, MN 55415 USA; 2 Genentech, Inc., South San Francisco, CA USA; 3 Division of Nephrology, Hennepin County Medical Center and Department of Medicine, University of Minnesota, Minneapolis, MN USA

**Keywords:** Dialysis bundled payment, End-stage renal disease, Vascular access management

## Abstract

**Background:**

We investigated whether implementation of the end-stage renal disease prospective payment system (ESRD PPS) was associated with changes in thrombolytic therapy use and other aspects of catheter management in hemodialysis (HD) patients.

**Methods:**

Using quarterly, period prevalent cohorts of patients undergoing maintenance HD with a catheter in the US Renal Data System (2008–2015), we studied rates of claims for within- and outside-HD-unit thrombolytic use, and thrombus/fibrin sheath removal, and rates of delayed HD treatment after ESRD PPS implementation, January 1, 2011. Associations between PPS implementation and change in trend of rates of each outcome were assessed using covariate-adjusted Poisson regression, using a piecewise linear function for quarter-time (with breakpoint at PPS implementation).

**Results:**

Among an average of 69,428 quarterly catheter users, rates of claims for within-HD-unit thrombolytic use declined from 236.6 (Q1–2008) to 81.4 (Q4–2012) per 100 person-years (*P* < 0.0001, PPS association with change in trend); rates of claims for thrombus/fibrin sheath removal procedures increased from 3.9 (Q1–2008) to 8.8 (Q3–2015) per 100 person-years (*P* = 0.0001, PPS association with change in trend). Rates of delayed HD treatment increased from 1.6 (Q2–2008) to 2.3 (Q3–2015) per patient-quarter, although PPS implementation was associated with a decrease in this rising trend (1.6% increase per quarter pre-PPS, 1.2% post-PPS; *P* < 0.0001, change in trend).

**Conclusions:**

After PPS implementation, thrombolytic use decreased and thrombus/fibrin sheath removal increased. The increasing trend in delayed HD treatment appeared to slow after PPS implementation, but delayed sessions continued to increase year over year for unclear reasons.

**Supplementary Information:**

The online version contains supplementary material available at 10.1186/s12882-020-02222-9.

## Background

In January 2011, the Centers for Medicare & Medicaid Services (CMS) implemented a bundled prospective payment system (PPS) for outpatient end-stage renal disease (ESRD) dialysis facilities (ESRD PPS) [1]. The ESRD PPS created a single comprehensive payment, adjusted for patient case-mix, for dialysis services, including injectable drugs and laboratory tests, which, when previously separately billable, comprised about 40% of total outpatient dialysis spending [1]. The effects of the ESRD PPS on dialysis care, particularly in relation to management of anemia and mineral metabolism [2–4], choice of modality and vascular access [5, 6], and major clinical events [7], have been widely studied.

Alteplase and other thrombolytic agents are among the injectable medications that were reimbursed separately from composite rate dialysis services in the pre-PPS period. Including these drugs in the PPS would, in theory, result in a reduction of their use in the outpatient dialysis unit. However, little is known about how implementation of the ESRD PPS affected thrombolytic use and other aspects of vascular access management, particularly among patients using central venous catheters for hemodialysis (HD) access. Elimination of separate reimbursement for thrombolytics in the HD unit could have unintended negative downstream consequences, including (i) more demand for thrombolytic use outside the HD unit, (ii) more invasive or costly declotting procedures (e.g., thrombectomy), and (iii) reduced quality of dialysis care (e.g., delayed HD treatment sessions). Using the United States Renal Data System (USRDS) database, we sought to investigate whether rates of these downstream consequences increased after implementation of the ESRD PPS, for HD patients using a catheter.

## Methods

### Data sources

We used standard analysis files from the 2007–2015 USRDS ESRD registry. The registry includes information on patient demographics, payer history, ESRD Medicare payment data, modality history, transplants, and the CMS Medical Evidence Report (form CMS-2728) and Death Notification (form CMS-2746). The payment data include diagnosis, procedure, and revenue center codes from all Part A institutional and Part B carrier claims, and Part D prescription drug claims. The research protocol was approved by the institutional review board at Hennepin Healthcare, and data use agreements between the Hennepin Healthcare Research Institute and the USRDS were in place.

### Study design

In this retrospective study, the primary analysis involved examining patterns of rates of catheter thrombosis management and delayed HD treatments in relation to implementation of the ESRD PPS. We created 31 quarterly period prevalent cohorts of maintenance HD-requiring ESRD patients who used a catheter for vascular access between January 1, 2008, and September 30, 2015. To be included in a quarterly cohort, patients had to have used a catheter for maintenance HD on at least 1 day during the quarter. We defined the index date as the first date of the quarter (for those using a catheter prior to the start of the quarter) or the first date of HD with a catheter (for those not using a catheter at the start of the quarter). Patients aged younger than 18 years on the index date, with a previous kidney transplant, or without continuous Medicare Parts A and B coverage for at least 3 months prior to the index date (or since HD initiation, if that occurred less than 3 months prior to the index date) were excluded. Thus, the cohorts included both prevalent and incident ESRD patients requiring maintenance HD. Patients using a catheter for HD (as described further below) in each quarterly cohort were followed from the index date until the earliest of: change in dialysis access or modality, kidney transplant, loss of Medicare coverage, death, or end of the quarter. In a secondary analysis, we used two cohorts (pre-PPS and post-PPS) to examine the association between location of thrombolytic administration (within vs. outside the HD unit) and delayed HD, as described further in Supplementary Methods.

### Identification of HD using a catheter

The modality history file, which provides a longitudinal record of modality changes, was used to determine HD start and end dates. The Medical Evidence Report provides information on the type of vascular access used for the first outpatient HD session, and on whether a maturing arteriovenous fistula (AVF) or graft (AVG) was present. Start dates for each period of catheter use were determined first based on the Medical Evidence Report and then by tunneled central venous catheter insertion procedures (using administrative codes in **Additional File 1: Table** **S1**) thereafter. End dates for each period of catheter use were determined using the earliest occurrence of: (i) tunneled central venous catheter removal, (ii) modality switch, (iii) 180 days with a maturing AVF or AVG or (iv) 24 months.

### Catheter thrombosis management

We identified procedures ostensibly related to catheter thrombosis management, including claims for thrombolytic use, mechanical thrombectomy, and fibrin sheath stripping (using administrative codes in **Additional File 1: Table** **S2**), during follow-up. Since the ESRD PPS changed the policy for reimbursement of injectable medications for outpatient dialysis facilities only, we identified thrombolytic use separately for claims within vs. outside the dialysis unit. We counted all procedures through follow-up, allowing for a maximum of one procedure of each type per day. Starting in 2013, CMS policy changed to discontinue reporting thrombolytic drugs on HD claims [8]; therefore, data from 2013 to 2015 were excluded from all models involving within-HD-unit thrombolytic claims. To investigate the potential cost implication of the PPS, we also identified total costs (Medicare payment amounts) from all Part A and B claims occurring in the 7-day period starting on the date of each catheter thrombosis procedure.

### Delayed HD treatment sessions

We hypothesized that, due to the elimination of separate reimbursement for injectable medications in the HD unit under the ESRD PPS, patients who previously would have received thrombolytics for catheter thrombosis directly in the dialysis unit would instead be referred elsewhere (e.g., a vascular access clinic), leading to delay in the scheduled HD treatment sessions. Thus, in addition to catheter thrombosis events, we also identified all delayed HD treatment sessions during follow-up. Patients receiving thrice-weekly HD follow a schedule with an expected sequence of 1-, 1-, and 2-day gaps between treatment sessions. Thus, we designated HD treatment sessions as being “delayed” if we saw a difference in the expected and observed gap. A detailed accounting of our methods for assessing delay can be found in **Supplementary Methods**.

### Baseline covariates

Patient demographics, primary cause of ESRD, and time since dialysis initiation (i.e., dialysis duration) were determined using the Medical Evidence Report. Medicare/Medicaid dual eligibility status, a proxy for socioeconomic disadvantage, was determined from the payer history file at each index date. As a marker of comorbid disease burden, we calculated the Liu comorbidity index [9]. The comorbid conditions comprising the comorbidity index were identified using the Medical Evidence Report and International Classification of Diseases, Ninth Revision, Clinical Modification (ICD-9-CM) diagnosis and V codes (**Additional File 1: Table** **S3**) from 1 or more inpatient or 2 or more outpatient billing claims occurring in the 3 months prior to the index date.

### Statistical analysis

For the primary analysis, baseline characteristics were described separately for each quarterly cohort. Event rates were estimated using Poisson regression, separately by each quarter, for each of the thrombosis management and delayed HD outcomes. To account for potential shifts in patient case-mix over time, the quarterly event rates were adjusted for all baseline covariates using a model-based method [10], using the cohort from the first quarter (Q1) of 2011 as the reference population. Bootstrap 95% confidence intervals (CI) were estimated using 1000 replicate samples.

Then, to evaluate the association between the ESRD PPS and change in trend of the rates of each outcome, we performed an interrupted time series analysis of the quarterly cohorts using segmented regression. This approach is particularly useful for retrospectively investigating the effects of a new intervention or policy with regard to longitudinal changes in clinical practice or patient outcomes [11, 12]. Specifically, we used covariate-adjusted Poisson regression with all quarterly cohorts modeled together. Each model included a quarter-time variable (values from 1 to 31) modeled as a piecewise linear function with two pieces: the first from Q1–2008 to Q4–2010, and the second from Q1–2011 to Q3–2015. The second term of the piecewise function was used to determine whether a change occurred in the trend of the quarterly event rate in the post-PPS period. Given that patients could appear across multiple quarterly cohorts, we used the generalized estimating equations method to account for this correlation. Also, we noted a seasonal pattern upon inspection of the quarterly rates of delayed HD. Thus, for the delayed HD model only, we additionally adjusted for seasonal effects using a pair of sine and cosine functions with a period length of 4 quarters.

Analogously, interrupted time series analyses were also conducted to evaluate the association between the ESRD PPS and change in trend of mean total Medicare costs in the 7 days following each catheter thrombosis procedure using covariate-adjusted segmented linear regression. Costs were expressed in 2015 U.S. dollars [13].

For the secondary analysis, we sought to investigate whether delayed HD would be more common among patients who received thrombolytics outside of the HD unit versus within the HD unit. In each of the relatively short time windows of 7 days before and 7 days after the date of thrombolytic administration, we estimated odds ratios and 95% CIs for the association between location of thrombolytic administration and 7-day delayed HD using covariate-adjusted logistic regression.

## Results

Construction of the cohorts for the primary analysis, consisting of HD patients using a catheter for vascular access between 2008 and 2015, is shown in **Additional File 1: Fig.** **S1**. Each of the 31 quarterly cohorts included an average of 69,428 patients. Patient characteristics for the Q1 cohort from each year are shown in Table 1. Across all cohorts, the mean age was 64.2 years, 48.6% were female, and 57.4% were white. Prevalence of covariates was generally similar across the quarterly cohorts, with a few exceptions. Prevalence of some comorbid conditions increased over time, including dysrhythmia (21.8% in Q1–2008 vs. 27.9% in Q1–2015) and other cardiac disease (26.7% in Q1–2008 vs. 31.4% in Q1–2015). Likewise, prevalence of patients in the highest category of the Liu comorbidity index (≥ 8) also increased (29.6% in Q1–2008 vs. 33.0% in Q1–2015).

**Table 1:** Patient characteristics across the quarterly cohorts (presenting only the first cohort in each year)

	Q1–2008	Q1–2009	Q1–2010	Q1–2011	Q1–2012	Q1–2013	Q1–2014	Q1–2015
Cohort size, *n*	71,215	72,262	70,861	70,046	69,689	71,442	68,693	66,502
Age category, years, %								
18–54	24.7	25.7	26.2	26.0	25.3	24.0	23.5	22.4
55–64	20.6	21.5	22.3	22.9	22.9	22.5	22.5	22.5
65–74	26.5	25.8	25.3	25.5	25.9	26.7	27.6	28.7
≥ 75	28.2	27.0	26.2	25.5	25.9	26.8	26.4	26.4
Female, %	49.1	49.4	49.3	48.8	48.5	48.3	47.9	47.4
Race, %								
White	58.0	56.9	56.7	56.4	56.9	58.0	58.3	59.0
Black	37.4	38.4	38.5	38.7	38.1	36.9	36.5	35.6
Other	4.6	4.7	4.8	4.9	5.0	5.1	5.3	5.3
Dual eligibility, %	46.2	46.9	47.3	48.7	48.5	45.7	49.1	48.4
Primary cause of ESRD, %								
Diabetes	47.5	47.3	47.3	47.3	47.1	47.2	47.5	47.8
Hypertension	29.6	29.6	29.8	30.0	30.1	30.7	31.0	31.0
GN or cystic kidney disease	9.7	10.1	10.0	9.7	9.6	9.1	8.8	8.4
Other	13.2	12.9	12.9	13.0	13.2	13.0	12.7	12.8
Dialysis vintage, %								
≤ 90 days	16.8	16.4	16.9	16.7	19.7	21.6	21.5	22.5
91-< 365 days	20.4	19.6	19.5	18.7	17.5	20.0	20.1	20.5
1-< 3 years	37.7	31.0	27.0	27.2	25.5	22.6	22.7	23.0
≥ 3 years	25.1	33.0	36.5	37.5	37.4	35.8	35.6	34.0
Liu comorbidity index, %								
0	9.3	9.7	9.7	9.2	8.9	8.3	8.4	8.1
1–4	35.5	35.4	35.4	34.3	34.4	34.5	34.9	34.6
5–7	25.6	25.3	24.9	24.3	24.1	24.3	24.2	24.3
≥ 8	29.6	29.6	30.0	32.3	32.7	32.9	32.4	33.0
Comorbid conditions, %								
Atherosclerotic heart disease	39.9	39.3	39.4	40.6	41.3	41.6	40.8	41.0
Congestive heart failure	51.3	50.7	50.2	50.7	50.5	50.3	49.5	50.5
Cerebrovascular disease	18.1	18.2	18.1	18.5	18.4	18.1	18.2	17.9
Peripheral vascular disease	33.6	33.4	33.5	34.8	34.3	33.9	33.9	34.1
Other cardiac disease	26.7	26.9	27.7	29.4	30.4	30.5	30.5	31.4
COPD	22.5	22.6	23.0	24.8	25.3	25.7	25.4	26.0
Gastrointestinal bleeding	5.9	5.7	5.6	6.1	6.4	6.0	5.9	5.8
Liver disease	5.6	5.7	5.4	6.2	6.5	6.2	5.8	5.2
Dysrhythmia	21.8	21.7	22.2	24.9	25.4	26.8	26.9	27.9
Cancer	9.5	9.4	9.5	9.6	9.8	9.7	9.8	9.8
Diabetes	64.6	64.4	65.0	66.5	67.3	67.9	67.7	68.1

COPD: chronic obstructive pulmonary disease; ESRD, end-stage renal disease; GN, glomerulonephritis

**Fig. 1:**
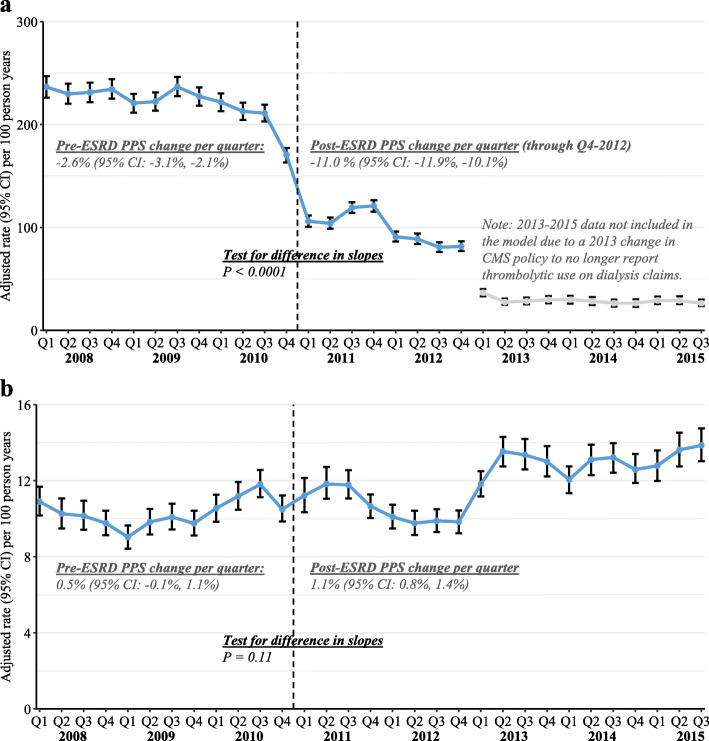
Quarterly rates and 95% confidence intervals for (a) within-HD-unit and (b) outside-HD-unit claims for thrombolytic use, standardized for age, sex, race, dual eligibility, primary cause of ESRD, ESRD duration, and Liu comorbidity index (using Q1–2011 as the reference). CI, confidence interval; ESRD, end-stage renal disease; PPS, prospective payment system

Quarterly, covariate-adjusted rates of claims for thrombolytics administered within and outside of the HD unit for patients using a catheter for HD are shown in Fig. 1. Across all quarters in the study period, thrombolytics where administered within the HD unit at a much higher rate (range 80.7 to 236.7 claims per 100 person-years) than outside the HD unit (9.0 to 13.9 per 100 person years). For within-HD-unit thrombolytic claims, which were directly affected by the revised payment model, rates declined by 2.6% (95% CI 2.1 to 3.1%) per quarter in the pre-PPS period and then declined by 11.0% (95% CI 10.1 to 11.9%) per quarter in the post-PPS period (*P* < 0.0001 for a change in trend post-PPS implementation). In contrast, for outside-HD-unit thrombolytic claims, which were not directly affected by the PPS, rates increased by 0.5% (95% CI − 0.1 to 1.1%) per quarter in the pre-PPS period and then increased by 1.1% (95% CI 0.8 to 1.4%) per quarter in the post-PPS period (*P* = 0.11 for post-PPS change in trend).

**Fig. 2:**
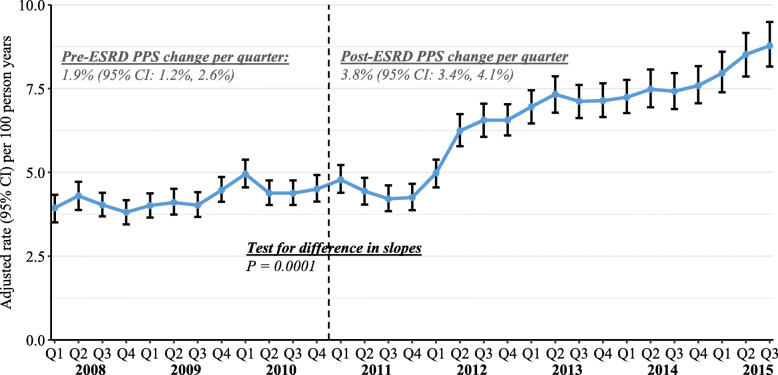
Quarterly rates and 95% confidence intervals for thrombus/fibrin sheath removal claims, standardized for age, sex, race, dual eligibility, primary cause of ESRD, ESRD duration, and Liu comorbidity index (using Q1–2011 as the reference). CI, confidence interval; ESRD, end-stage renal disease; PPS, prospective payment system

Quarterly, covariate-adjusted rates of claims for more invasive catheter management interventions, namely, thrombus/fibrin sheath removal, are shown in Fig. 2. Thrombus/fibrin sheath removal occurred less frequently than all other interventions, with rates ranging from 3.8 to 8.8 claims per 100 person-years across all quarters. Rates increased by 1.9% (95% CI 1.2 to 2.6%) per quarter in the pre-PPS period, and by 3.8% (95% CI 3.4 to 4.1%) per quarter in the post-PPS period (*P* = 0.0001 for post-PPS change in trend).

In **Additional File 1: Figs.** **S2** and **S3**, we present quarterly, covariate-adjusted mean total Medicare costs occurring in the 7 days after each thrombolytic administration, separately for claims occurring within and outside of the HD unit, and in the 7 days after each thrombus/fibrin sheath removal procedure. For each intervention type, costs appeared to increase over time in the pre-PPS period and then decrease over time in the post-PPS period (*P* < 0.0001 for post-PPS change in trend).

**Fig. 3:**
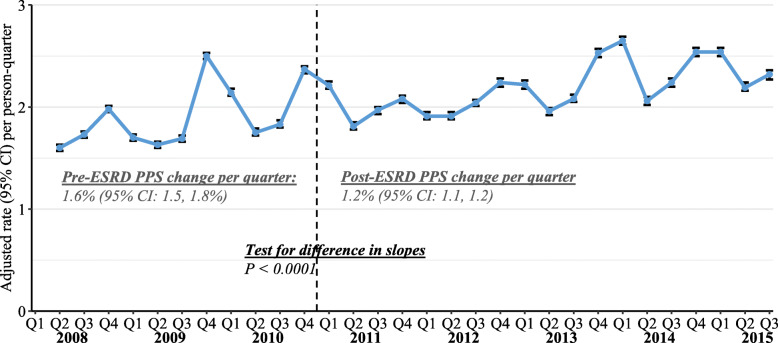
Quarterly rates and 95% confidence intervals of delayed HD sessions, standardized for age, sex, race, dual eligibility, primary cause of ESRD, ESRD duration, and Liu comorbidity index (using Q1–2011 as the reference). CI, confidence interval; ESRD, end-stage renal disease; PPS, prospective payment system

Quarterly, covariate-adjusted rates of delayed HD sessions, a quality-of-care marker potentially affected by the revised payment model, are shown in Fig. 3. Across the study period, delayed HD occurred at a rate ranging from 1.6 to 2.7 days per person per quarter. Notably, delayed HD generally appeared to occur more frequently in the last quarter of each year, possibly a result of scheduling alterations around major US holidays. Delayed HD rates increased by 1.6% (95% CI 1.5 to 1.8%) per quarter in the pre-PPS period, and continued increasing, but to a slightly lesser degree, by 1.2% (95% CI 1.1 to 1.2%) per quarter in the post-PPS period (*P* < 0.0001 for post-PPS change in trend).

Results for the secondary analysis, in which we created two cohorts (pre- and post-PPS) restricted to only patients receiving thrombolytics, are shown in Table 2. In the pre-PPS period, receiving thrombolytics outside, versus within, the HD unit was associated with 2.11-fold (95% CI 1.91–2.32) and 1.30-fold (95% CI 1.16–1.47) greater odds of experiencing one or more delayed HD sessions in the 7 days before and after the date of thrombolytic administration, respectively. In the post-PPS period, the analogous odds ratios for delayed HD were 2.02 (95% CI 1.82–2.26) and 1.21 (95% CI 1.07–1.38), respectively.

**Table 2:** Odds ratios and 95% confidence intervals for the association of location of first thrombolytic use with delayed HD

	Any delayed HD						
		In 7 days before^*^ thrombolytics			In 7 days after thrombolytics		
Location of thrombolytic administration	*n* patients	*n* events	%	OR (95% CI)	*n* events	%	OR (95% CI)
Pre-PPS (2008–2010)							
Within-HD-unit	39,567	2417	6.1	1 (Ref)	2359	6.0	1 (Ref)
Outside-HD-unit	4467	587	13.1	2.11 (1.91–2.32)	353	7.9	1.30 (1.16–1.47)
Post-PPS (2011–2012)							
Within-HD-unit	18,509	1284	6.9	1 (Ref)	1252	6.8	1 (Ref)
Outside-HD-unit	4071	579	14.2	2.02 (1.82–2.26)	340	8.4	1.21 (1.07–1.38)

Models are adjusted for age, sex, race, dual eligibility, primary cause of ESRD, ESRD duration, and Liu comorbidity index

^*^This 7-day period includes the date of thrombolytic administration

CI, confidence interval; ESRD, end-stage renal disease; HD, hemodialysis; OR, odds ratio; PPS, prospective payment system

## Discussion

This study examined practice patterns, before and after implementation of the ESRD PPS, related to HD catheter thrombosis management in a series of large national cohorts of Medicare beneficiaries receiving maintenance HD. We found that implementation of the PPS was associated with several shifts in care, including a decline in within-HD-unit thrombolytic claims and an increase in thrombus/fibrin sheath removal claims. Although we did not find evidence of an association of the PPS implementation with a shift in outside-HD-unit thrombolytic claims, claims did appear to increase starting in 2013. We also found that the rate of delayed HD sessions increased over the entire study period; implementation of the PPS was associated with a reduction, but not a reversal, of this increasing trend. Finally, among patients receiving thrombolytic therapy, drug administration outside, vs. within, the HD unit was associated with increased risk of delayed HD treatment in both the pre-PPS and post-PPS periods.

We observed a decline, by approximately 55%, in the rate of thrombolytic use in the dialysis unit, and the coincident 56% increase in the rate of thrombus/fibrin sheath removal procedures (i.e., those relying on a skilled interventional specialist—nephrologist, radiologist, or surgeon), comparing the post-PPS and the pre-PPS periods. A possible explanation for these findings is that, under the PPS, a payment model that eliminated separate reimbursement for injectable medications, some cases of catheter thrombosis, which previously may have been resolved directly within the HD unit by administering thrombolytic therapy, were more likely to be shifted to another setting (e.g., a vascular access center or other radiology suite) to perform a more intensive procedure. Other studies have reported similar post-PPS shifts in care in maintenance HD patients. For example, with respect to anemia management, a decline in use of erythropoiesis-stimulating agents (ESAs) coincided with an increase in red blood cell transfusions after PPS implementation (and an updated ESA drug label) in 2011 [2, 3, 14].

Furthermore, many of the additional transfusions required care in more intensive settings (i.e., inpatient hospital and emergency department) [15]. Also, it is worth noting that thrombolytic use in the dialysis unit began declining in the quarter prior to PPS implementation (19% decrease between Q3 and Q4 of 2010). Since CMS published the final rule of PPS regulations in August 2010 [1], the decline likely represents an anticipatory reaction, similar to the decline in use of ESAs in the months prior to PPS implementation [3], and the decline in the rate of hospital readmissions in the 2 years preceding the start of the Hospital Readmissions Reduction Program in October 2012 [16].

Along with the PPS-associated decrease in within-HD-unit thrombolytic use and increase in thrombus/fibrin sheath removal procedures, we also expected to observe clear PPS-associated shifts toward increased use of thrombolytics outside the HD unit. While thrombolytic use outside the HD unit appeared to increase by nearly 40% from 2012 to 2013, we found no clear evidence that this was associated with the implementation of the PPS given that the increase began to occur several years after PPS implementation. One possibility is that PPS implementation and the apparent increase in non-HD-unit use of thrombolytics are entirely unrelated. A second explanation is that thrombolytic use within the HD unit has slowly become more disfavored over time, perhaps due to cost constraints or the reimbursement environment. Anecdotal experience suggests that many HD units order fewer monthly doses of thrombolytics than in years past, creating a less liberal environment for its use than before. A third explanation is that mechanical interventions, as opposed to use of thrombolytics, may be favored as more definitive and financially rewarding methods for resolving catheter malfunction by interventionalists who treat dialysis patients referred outside the HD unit, even if thrombolytics may have provided an adequate temporary solution. Future studies examining this trend in present and future years may be in a position to offer more insights into this issue.

It is also important to consider the economic implications of the PPS. For each catheter thrombosis intervention occurring in our study period, we examined the total Medicare costs occurring over a weeklong period starting on the date of the intervention. We found that there was a PPS-associated shift towards decreased costs. We note that these results demonstrate only how costs shifted among patients receiving the *same* types of procedures over time, but not how overall costs for treating catheter thrombosis events may have changed. Unfortunately, we were unable to investigate the latter question due to the limited specificity among ICD-9-CM diagnosis codes for identifying catheter thrombosis events directly. We speculate that, with the shifts towards less frequent in-center thrombolytic administrations and more frequent mechanic clot removal procedures, total Medicare costs for the treatment of catheter thrombosis events have increased in the post-PPS period. However, this hypothesis would need to be investigated using another data source.

The findings regarding trends in delayed treatment were somewhat nuanced. Delayed HD sessions, perhaps surprisingly, continued to increase throughout the study period, although the PPS implementation was associated with a slowing of this increasing trend. We observed nearly a 50% increase in delayed HD, from 1.6 sessions per patient per quarter in Q2 of 2008 to 2.3 sessions per patient per quarter by Q3 of 2015, which is similar in magnitude to the 7.1 days of missed treatment per patient per year reported from a large cohort of HD patients from a national dialysis chain in 2005–2009 [17]. We find this potential increasing trend of delayed HD (which has also been reported in other US HD cohorts [18]) concerning, particularly since missed HD treatment is associated with greater risk of numerous adverse outcomes, including hospitalization and death [17, 19]. Future investigations should determine whether implementation of newer care models that incentivize coordinated ESRD care, such as the 2015 CMS Comprehensive ESRD Care Model and ESRD seamless care organizations, has led to reductions in delayed HD.

In secondary analyses, we found evidence that thrombolytic administration outside, as opposed to within, the HD unit was associated with greater risk of delayed HD in the pre- and post-PPS periods. Our rationale for undertaking this comparison was that thrombosis may lead to delayed HD treatment because (i) thrombolytics are not immediately available within the HD unit, (ii) thrombolytics are available, but the time required for instillation delays treatment until the following day, or (iii) thrombolytics are available, but instillation is unsuccessful and a more invasive declotting procedure is required. Admittedly, disentangling the direction of this association using observational data is somewhat complicated, as patients with access thrombosis may experience delayed treatment either before or after receiving thrombolytics. Because we found that the location of administration was associated with delay in the week before thrombolytic administration, we suspect that access to thrombolytics in the HD unit may decrease risk for treatment delays. Nevertheless, we cannot rule out the possibility that this association is confounded by occlusion history, whereby patients with prior access complications are more likely to be referred outside of the HD unit to treat the thrombosis.

One potential therapeutic approach to decreased use and/or availability of thrombolytics as “rescue” agents in the dialysis unit might be prophylactic or “preemptive” use of such agents on a regular schedule. A trial conducted a decade ago suggested that once-weekly use of recombinant tissue plasminogen activator as a catheter locking solution (in place of heparin) is associated with decrease risk of catheter malfunction and bacteremia) [20]. However, for this to be an effective strategy in the U.S., the reimbursement structure likely would need to be altered to accommodate routine use of thrombolytics as catheter locking agents.

This study has several limitations. First, it can be challenging to assess the impact of a policy change using observational data. Our findings may be biased due to other unaccounted changes, such as with respect to patient characteristics or treatment practices, between the pre- to post-PPS periods. We generally observed similar patient characteristics across the quarterly cohorts, and standardized or adjusted for these covariates in our models, but residual confounding is still a possibility. Second, start and end dates for patient follow-up were defined, in part, on procedure codes for insertion, replacement, and removal of tunneled central venous catheters, but not using codes for non-tunneled catheters, which we could not be sure were specific to the vascular access for HD. Non-tunneled (temporary) catheters are used relatively infrequently for maintenance HD, at least in the US, so we expect this should have a minimal impact on our findings. Third, given our use of administrative claims data, we were limited in our ability to fully characterize catheter thrombosis, both with regard to its exact date of occurrence and severity. For this reason, we were able to describe only rates of thrombosis-related care in a broad sense, but not rates of thrombosis itself. Finally, our findings are limited to the ESRD population with Medicare Part A and B (fee-for-service) coverage.

## Conclusions

In summary, the ESRD PPS implementation was associated with an approximate 55% decrease in claims for thrombolytic use in the dialysis unit and a 56% increase in claims for thrombus and fibrin sheath removal procedures. Delayed HD treatment rates increased across the study period, although PPS implementation was associated with a slowing of this trend. Use of thrombolytics outside, as opposed to within, the dialysis unit, may be associated with increased risk of HD treatment delay. Future work should further explore trends and determinants of delayed HD treatment beyond 2015.

## Supplementary Information

**Additional file 1: Table S1.** Administrative codes used to identify catheter use for hemodialysis. **Table S2.** Administrative codes used to identify catheter management-related events. **Table S3.** ICD-9-CM codes used to define the comorbid conditions comprising the Liu comorbidity index. **Fig. S1.** Construction of the quarterly cohorts (presenting only the first cohort in each year). HD, hemodialysis. **Fig. S2.** Quarterly mean total Medicare costs in the 7-day period starting from the date of each claim for thrombolytic use, separately for (a) within-HD-unit and (b) outside-HD-unit administrations. Costs are presented with 95% confidence intervals and are standardized for age, sex, race, dual eligibility, primary cause of ESRD, ESRD duration, and Liu comorbidity index (using Q1–2011 as the reference). CI, confidence interval; ESRD, end-stage renal disease, PPS, prospective payment system. **Fig. S3.** Quarterly mean total Medicare costs in the 7-day period starting from the date of each claim for a thrombus/fibrin sheath removal procedure. Costs are presented with 95% confidence intervals and are standardized for age, sex, race, dual eligibility, primary cause of ESRD, ESRD duration, and Liu comorbidity index (using Q1–2011 as the reference). CI, confidence interval; ESRD, end-stage renal disease, PPS, prospective payment system. **Supplemental Methods.**

## Data Availability

The datasets analyzed during the current study were obtained from the United States Renal Data System (USRDS). These data are publicly available and free of charge from the USRDS Coordinating Center (https://usrds.org). Data are available from the USRDS.

## Abbreviations

AVF: arteriovenous fistula
AVG: arteriovenous graft
CI: confidence interval
CMS: Centers for Medicare & Medicaid Services
ESA: erythropoiesis-stimulating agent
ESRD: end-stage renal disease
HD: hemodialysis
ICD-9-CM: International Classification of Diseases, Ninth Revision, Clinical Modification
PPS: prospective payment system
USRDS: United States Renal Data System
