# Correction to: Changes in hemodialysis catheter management after introduction of the end-stage renal disease prospective payment system

**DOI:** 10.1186/s12882-021-02300-6

**Published:** 2021-03-15

**Authors:** Nicholas S. Roetker, Haifeng Guo, Marquita R. Decker-Palmer, Yi Peng, James B. Wetmore

**Affiliations:** 1 Chronic Disease Research Group, Hennepin Healthcare Research Institute, 701 Park Ave., Suite S2.100, Minneapolis, MN 55415 USA; 2 Genentech, Inc., South San Francisco, CA USA; 3 Division of Nephrology, Hennepin County Medical Center and Department of Medicine, University of Minnesota, Minneapolis, MN USA

**Correction to: BMC Nephrol (2021) 22:8**

**https://doi.org/10.1186/s12882-020-02222-9**

Following publication of the original article [1], the authors would like to add the **Disclaimer** language and to update the **Availability of data and materials** section as follows:

**Disclaimer**

The data reported here have been supplied by the United States Renal Data System (USRDS). The interpretation and reporting of these data are the responsibility of the author(s) and in no way should be seen as an official policy or interpretation of the U.S. government.

**Availability of data and materials**

The data used for this study are available, free of charge, to any entity that enters into a Data Use Agreement with the USRDS. Requests may be emailed to USRDS@usrds.org.

The original article has been corrected.
