# Supplementary material for: Changes in hemodialysis catheter management after introduction of the end-stage renal disease prospective payment system
Source: BMC Nephrol. 2021 Jan 6;22:8. doi: 10.1186/s12882-020-02222-9 (PMC7788942; doi:10.1186/s12882-020-02222-9)
Supplement: Supplementary file 1 — Additional file 1: Table S1. Administrative codes used to identify catheter use for hemodialysis. Table S2. Administrative codes used to identify catheter management-related events. Table S3. ICD-9-CM codes used to define the comorbid conditions comprising the Liu comorbidity index. Fig. S1. Construction of the quarterly cohorts (presenting only the first cohort in each year). HD, hemodialysis. Fig. S2. Quarterly mean total Medicare costs in the 7-day period starting from the date of each claim for thrombolytic use, separately for (a) within-HD-unit and (b) outside-HD-unit administrations. Costs are presented with 95% confidence intervals and are standardized for age, sex, race, dual eligibility, primary cause of ESRD, ESRD duration, and Liu comorbidity index (using Q1–2011 as the reference). CI, confidence interval; ESRD, end-stage renal disease, PPS, prospective payment system. Fig. S3. Quarterly mean total Medicare costs in the 7-day period starting from the date of each claim for a thrombus/fibrin sheath removal procedure. Costs are presented with 95% confidence intervals and are standardized for age, sex, race, dual eligibility, primary cause of ESRD, ESRD duration, and Liu comorbidity index (using Q1–2011 as the reference). CI, confidence interval; ESRD, end-stage renal disease, PPS, prospective payment system. Supplemental Methods. [file 12882_2020_2222_MOESM1_ESM.docx]

**Additional File**

**Additional File Table S1.** Administrative codes used to identify catheter use for hemodialysis

**Additional File Table S2.** Administrative codes used to identify catheter management-related events

**Additional File Table S3.** ICD-9-CM codes used to define the comorbid conditions comprising the Liu comorbidity index

**Additional File Figure S1.** Construction of the quarterly cohorts (presenting only the first cohort in each year). HD, hemodialysis

**Additional File Figure S2.** Quarterly mean total Medicare costs in the 7-day period starting from the date of each claim for thrombolytic use, separately for (a) within-HD-unit and (b) outside-HD-unit administrations. Costs are presented with 95% confidence intervals and are standardized for age, sex, race, dual eligibility, primary cause of ESRD, ESRD duration, and Liu comorbidity index (using Q1-2011 as the reference). CI, confidence interval; ESRD, end-stage renal disease, PPS, prospective payment system.

**Additional File Figure S3.** Quarterly mean total Medicare costs in the 7-day period starting from the date of each claim for a thrombus/fibrin sheath removal procedure. Costs are presented with 95% confidence intervals and are standardized for age, sex, race, dual eligibility, primary cause of ESRD, ESRD duration, and Liu comorbidity index (using Q1-2011 as the reference). CI, confidence interval; ESRD, end-stage renal disease, PPS, prospective payment system.

**Supplemental Methods**

**Additional File Table S1.** Administrative codes for hemodialysis vascular access

| Event | Code |
| --- | --- |
| Tunneled central venous catheter insertion | CPT: 36558, 36565, 36581 |
| Tunneled central venous catheter removal | CPT: 36589 |
| Placement of an AVF or AVG | CPT: 36818, 36819, 36820, 36821, 36825, 36830, 36831, 36832, 36833, or by form CMS-2728 |

AVF, arteriovenous fistula; AVG, arteriovenous graft; CMS, Centers for Medicare & Medicaid Services; CPT, Current Procedural Terminology.

**Additional File Table S2.** Administrative codes used to identify catheter management-related events

| Event | Code | Notes |
| --- | --- | --- |
| Thrombolytic use  (on any claim type) | CPT 36593 |  |
| Thrombolytic use (on outpatient dialysis claim) | HCPCS: J3364, J3365, J0350, J2993, J2995, J2997, J3101 |  |
| Thrombolytic use (on any claim type besides outpatient dialysis) | HCPCS: J3364, J3365, J0350, J2993, J2995, J2997, J3101;  ICD-9-PCS: 99.10 | To ensure the thrombolytic use was related to the HD access, we also required one of the following codes to appear on the same day:  ICD-9-CM diagnosis: 996.1, 996.73, 996.74; CPT: 36558, 36565, 36581, 36589 |
| Thrombus/fibrin sheath removal (on any claim type) | CPT: 36595, 36596, 75901, 75902 |  |

CPT, Current Procedural Terminology; HCPCS, Healthcare Common Procedure Coding System; ICD-9-CM, International Classification of Diseases, Ninth Revision, Clinical Modification.

**Additional File Table S3.** ICD-9-CM codes used to define the comorbid conditions comprising the Liu comorbidity index

| Comorbid condition | ICD-9-CM diagnosis codes | ICD-9-CM V codes |
| --- | --- | --- |
| ASHD | 410-414 | V45.81, V45.82 |
| Congestive heart failure | 398.91, 422, 425, 428, 402.X1, 404.x1, 404.x3 | V42.1 |
| Cerebrovascular accident/ transient ischemic attack | 430-438 |  |
| Peripheral vascular disease | 440-444, 447, 451-453, 557 |  |
| Other cardiac disease | 420-421, 423-424, 429, 785.0-785.3 | V42.2, V43.3 |
| Chronic obstructive pulmonary disease | 491-494, 496, 510 |  |
| Gastrointestinal bleeding | 456.0-456.2, 530.7, 531-534, 569.84, 569.85, 578 |  |
| Liver disease | 570, 571, 572.1, 572.4, 573.1-573.3 | V42.7 |
| Dysrhythmia | 426-427 | V45.0, V53.3 |
| Cancer | 140-172, 174-208, 230-231, 233-234 |  |
| Diabetes | 250, 357.2, 362.0x, 366.41 |  |

ICD-9-CM, International Classification of Diseases, Ninth Revision, Clinical Modification.

**Additional File Figure S1.** Construction of the quarterly cohorts (presenting only the first cohort in each year). HD, hemodialysis.

**Additional File Figure S2.** Quarterly mean Medicare expenditures and 95% confidence intervals in the 7 days following (a) within-HD-unit and (b) outside-HD-unit claims for thrombolytic use, standardized for age, sex, race, dual eligibility, primary cause of ESRD, ESRD duration, and Liu comorbidity index (using Q1-2011 as the reference). CI, confidence interval; ESRD, end-stage renal disease, PPS, prospective payment system.

**
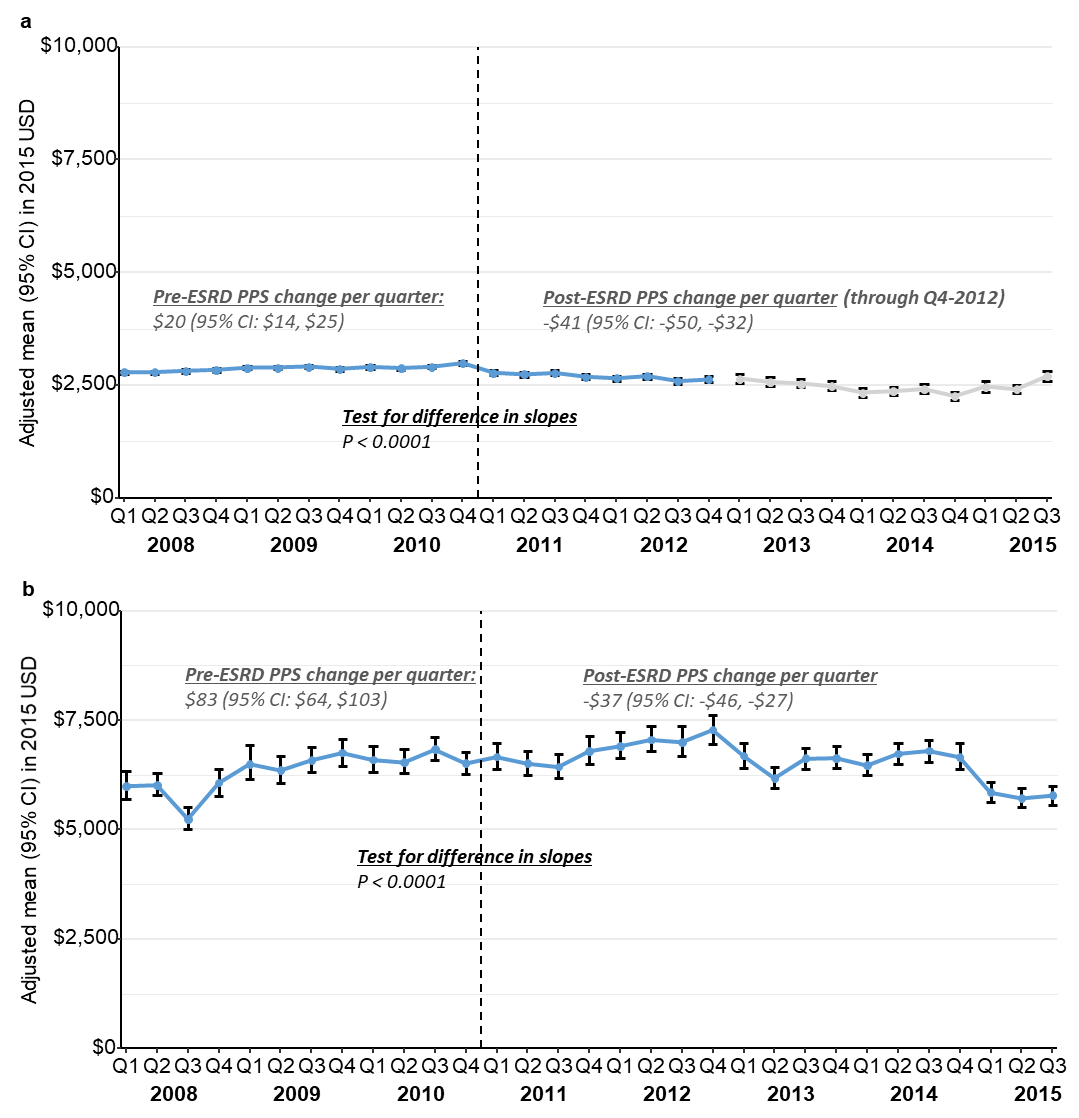
**

**Additional File Figure S3.** Quarterly mean Medicare expenditures and 95% confidence intervals in the 7 days following claims for thrombus/fibrin sheath removal, standardized for age, sex, race, dual eligibility, primary cause of ESRD, ESRD duration, and Liu comorbidity index (using Q1-2011 as the reference). CI, confidence interval; ESRD, end-stage renal disease, PPS, prospective payment system.

**
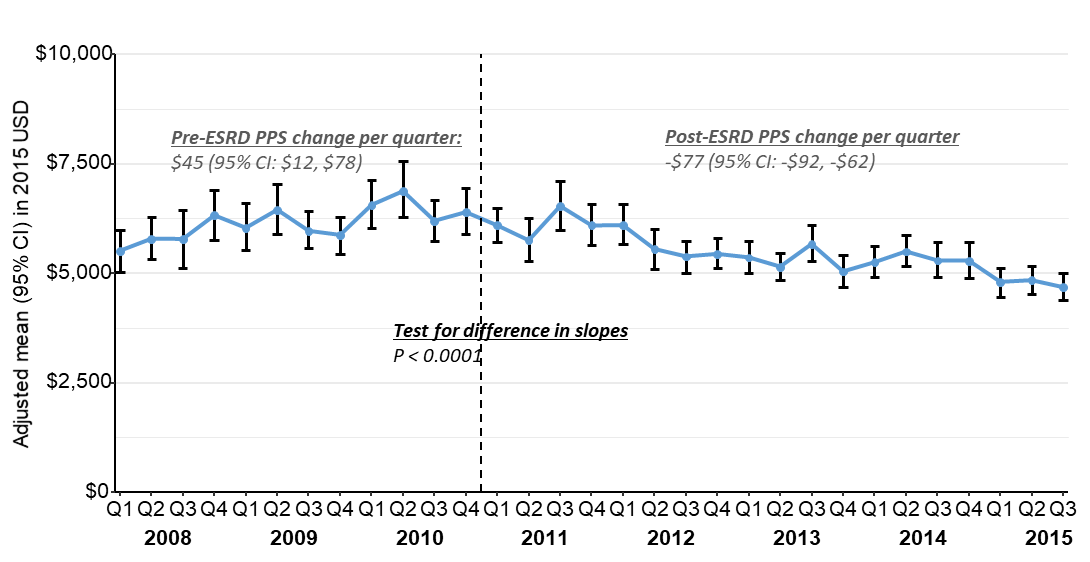
**

**Supplementary Methods**

*Study design for secondary analysis*

We created two cohorts of patients: one pre-PPS (2008-2010) and the other post-PPS (2011-2012; we excluded 2013-2015 due to a CMS policy change to discontinue reporting thrombolytic drugs on HD claims). To be included in one of the cohorts, patients had to be eligible for at least one quarterly cohort for the primary analysis and have a claim for thrombolytic use. Only the first instance of thrombolytic use in each of the pre- or post-PPS periods was considered. Then, we identified delayed HD within a 7-day day period before and after the date of thrombolytic use. We also identified catheter replacements, but only in the 7-day period after the date of thrombolytic use.

*Delayed HD sessions*

In each quarter, delayed HD was defined only among the subset of patients receiving thrice-weekly HD, using the previous quarter to determine the HD schedule (thus, the delayed HD outcome was assessed starting only in Q2-2008). In a given quarter, we included only patients who (i) used a catheter for HD prior to the current quarter, (ii) had at least three HD sessions in the prior quarter, with at least one in the last month of the quarter, (iii) dialyzed exactly three times in a majority of weeks in the prior quarter, and (iv) had at least one HD session in the current quarter.
 For example, a patient on a Monday-Wednesday-Friday schedule who dialyzes on Monday-Thursday-Friday would be assigned a 1-day delay due to delaying the Wednesday session by 1 day. We identified all delays of 1 to 3 days. We used 3 days as the maximum delay, even if it appeared to be longer, because we thought it was not reasonable to assume a patient would skip maintenance HD for longer than 3 days. These patients may actually have received HD through another mechanism (e.g., secondary insurance) for which we have no record. We did not assign delay during hospitalizations since we had no record of inpatient HD sessions. The one exception was for patients with a diagnosis code for a vascular access complication (International Classification of Diseases, Ninth Revision, Clinical Modification [ICD-9-CM] 996.1, 996.73, or 996.74), in which case we assigned a delay of 1 day, regardless of the length of the hospitalization. Under this framework, we assumed the access complication (i.e., clot) contributed to the patient being unable to undergo his or her regular outpatient HD session, and the need for hospitalization represents a form of delay.
